# Supplementary material for: The Relationship between the Unmet Needs of Chinese Family Caregivers and the Quality of Life of Childhood Cancer Patients Undergoing Inpatient Treatment: A Mediation Model through Caregiver Depression
Source: Int J Environ Res Public Health. 2022 Aug 17;19(16):10193. doi: 10.3390/ijerph191610193 (PMC9408546; doi:10.3390/ijerph191610193)
Supplement: Supplementary file 1 [file ijerph-19-10193-s001.zip › ijerph-1857560-supplementary.pdf]

Table S1. Detailed Multiple linear regression models among the Unmet Needs and Depression of Caregivers and the QoL of Care-recipients (N=286).

Characteristics	Model 1				Model 2				Model 3				Model 4			
	B (95% CI)	P-value	R^2	F	B (95% CI)	P-value	R^2	F	B (95% CI)	P-value	R^2	F	B (95% CI)	P-value	R^2	F
Main Terms			0.1484	3.37^{***}			0.2193	5.44^{***}			0.1944	4.67^{***}			0.2110	4.81^{***}
QoL	1.00								1.00				1.00			
Depression					1.00				-1.03(-1.37 – 0.69)	<0.001			-0.86(-1.23 – -0.49)	<0.001		
Unmet Needs	-0.21(-0.31 - -0.12)	<0.001			0.11(0.08 – 0.14)	<0.001							-0.12(-0.22 – -0.02)	0.018*		
Controls																
**Patient Characteristics**																
Age	0.07（-0.98-1.12）	0.896			-0.43（-0.76—0.10）	0.012*			-0.34（-1.37—0.70）	0.522			-0.30（-1.32—0.73）	0.568		
Gender																
Male	1.00				1.00				1.00				1.00			
Female	-3.16(-8.23 – 1.91)	0.220			-0.78(-2.38 – 0.82)	0.338			-3.58(-8.51 – 1.36)	0.155			-3.83(-8.73 – 1.06)	0.124		
Education																
Educated	1.00				1.00				1.00				1.00			
Uneducated	2.25(-4.38 – 8.87)	0.518			0.80(-1.28 – 0.82)	0.760			3.34(-3.10 – 9.78)	0.308			2.94(-3.46 – 9.33)	0.366		
Single Child																
Yes	1.00				1.00				1.00				1.00			
No	3.38(-2.73 – 9.49)	0.216			-0.94(-2.86 – 0.99)	0.340			2.98(-2.96 – 8.92)	0.324			2.57(-3.32 – 8.47)	0.391		

Cancer Type								
Leukemia	1.00		1.00		1.00		1.00	
Solid Tumor	-10.20(-21.00 – 0.60)	0.064	1.94(-1.47 – 5.34)	0.263	-8.23(-18.75 – 2.30)	0.125	-8.53(-18.97 – 1.91)	0.109
**Caregiver Characteristics**								
Age	-0.07 (-0.56-0.41)	0.775	0.02（-0.13-0.18)	0.750	-0.09 (-0.56-0.38)	0.694	-0.05 (-0.52-0.42)	0.836
Gender								
Male	1.00		1.00		1.00		1.00	
Female	3.08(-2.53 – 8.69)	0.388	0.72(-1.05 – 2.49)	0.425	3.96(-1.49 – 9.42)	0.154	3.70(-1.72 – 9.11)	0.180
Education								
Compulsory Education	1.00		1.00		1.00		1.00	
High school and above	0.69(-4.56 – 5.94)	0.260	-2.19(-3.84 – 0.53)	0.010*	-2.16(-7.27 – 2.94)	0.404	-1.19(-6.31 – 3.93)	0.648
Surgery								
Yes	1.00		1.00		1.00		1.00	
No	-3.35(-13.26 – 6.56)	0.507	-0.81(-3.94 – 2.31)	0.609	-4.72(-14.35 – 4.91)	0.335	-4.05(-13.61 – 5.52)	0.406
Radiation therapy								
Yes	1.00		1.00		1.00		1.00	
No	2.21(-5.56 – 9.98)	0.576	1.85(-0.61 – 4.30)	0.139	4.14(-3.44 – 11.73)	0.283	3.80(-3.73 – 11.33)	0.321
Number of chemotherapy								
≤10	1.00		1.00		1.00		1.00	

>10	-6.47(-11.81 – -1.13)	0.018*	0.03(-1.65 – 1.72)	0.968	-6.41(-11.60 – -1.22)	0.016*	-6.44(-11.59 – -1.29)	0.014*
Recurrence								
Yes	1.00		1.00		1.00		1.00	
No	5.33(-2.84 – 13.50)	0.200	0.38(-2.20 – 2.96)	0.771	5.57(-2.37 – 13.52)	0.168	5.66(-2.22 – 13.54)	0.158
Metastasis								
Yes	1.00		1.00		1.00		1.00	
No	4.26(-2.88 – 13.50)	0.241	-0.60(-2.86 – 1.65)	0.597	3.90(-3.05 – 10.85)	0.270	3.74(-3.15 – 10.63)	0.286

***P-value <0.001,*P-value <0.05
